# Orthodontic patients’ oral hygiene compliance by utilizing a smartphone application (Brush DJ): a randomized clinical trial

**DOI:** 10.1038/s41405-020-00050-5

**Published:** 2020-11-20

**Authors:** Homa Farhadifard, Sepideh Soheilifar, Maryam Farhadian, Hadi Kokabi, Anahita Bakhshaei

**Affiliations:** 1 Department of Orthodontics, School of Dentistry, Hamadan University of Medical Sciences, Hamadan, Iran; 2 Department of Biostatistics, School of Public Health and Research Center for Health Sciences, Hamadan University of Medical Sciences, Hamadan, Iran; 3 Periodontology Department, Hamadan University of Medical Sciences, Hamadan, Iran

**Keywords:** Dentistry, Oral hygiene

## Abstract

Considering the widespread use of smartphones and their applications (apps), as well as the undeniable role of reminders and apps in behavioral interventions, this study aimed to assess the efficacy of a smartphone app (Brush DJ) for oral hygiene compliance of patients with fixed orthodontic appliances. In this randomized clinical trial, 120 patients between 15 to 25 years who had just started fixed orthodontic treatment were randomly divided into two groups (*n* = 60). Control patients received conventional oral hygiene instruction, while patients in the intervention group were asked to use the Brush DJ smartphone app, after receiving conventional oral hygiene instruction. The plaque index (PI) and gingival index (GI) were measured at baseline (T0), and at 4 weeks (T1), 8 weeks (T2) and 12 weeks (T3) after the onset of study. A questionnaire was given to all patients to assess the frequency and duration of tooth brushing per day, and the frequency of app usage and reminder noticing in the intervention group. Improvements in PI and GI were noted in the intervention group; while these parameters increased in the control group. Significant differences were noted in PI and GI changes between the two groups (*p* < 0.001). Brushing frequency and duration were positively correlated with app usage during the follow-up period. Ultimately, we believe that smartphone apps, as motivators and reminders, can greatly help in improving the orthodontic patients’ oral hygiene compliance, especially in adolescents

## Introduction

Dental alignment facilitates oral hygiene, and can consequently decrease the risk of caries and periodontal disease.^1^ However, bacterial plaque accumulation around orthodontic brackets can lead to development of white spot lesions during orthodontic treatment since fixed orthodontic appliances interfere with dental plaque removal.^2,3^ Bacterial accumulation around the teeth can result in gingival inflammation, bleeding, oedema, and changes in gingival morphology and gingival crevicular fluid.^3^ Mechanical plaque removal can decrease the risk of chronic gingivitis, but many orthodontic patients do not have sufficient motivation for efficient dental plaque removal; this problem is more obvious in adolescent orthodontic patients.^4,5^

Previous studies have revealed a rapid decline in oral hygiene status after the first session of orthodontic treatment followed by a rapid improvement at 5 months following the treatment onset.^6^ However, the poorest oral hygiene compliance has been reported at the end of treatment by some other studies.^7^ Thus, achieving acceptable and sustainable oral hygiene remains a challenge in orthodontic patients.^3^

Dentists often have difficulty in providing their patients with adequate and efficient oral hygiene instructions.^8^ Thus, telemedicine, which incorporates mobile technology, can now be for considered for this purpose since it can enable an excellent communication between patients and dentists.^9^

Some particular features of smartphones that make them suitable for behavioral interventions include: (1) they are portable devices that are extremely popular among people specially adolescents. (2) Smartphone applications (apps) are a more economical and favorable way of intervention. (3) The capability of smartphones for a convenient connection facilitates the distribution of health-related information and provision of behavioral interventions.^10^

Therefore, considering the widespread use of smartphones and apps in diverse fields by the younger generation, as well as the undeniable role of reminders and apps in behavioral interventions, use of smartphone apps for this purpose is understandable. Hence, in this study, we utilized a mobile health technology, the Brush DJ app (https://www.nhs.uk/apps-library/brush-dj/), to manage the orthodontic patients’ oral hygiene frequency and duration. The main objective of this study was to assess the efficacy of a smartphone app, compared with the conventional method, to improve the oral hygiene compliance of patients with fixed orthodontic appliances.

## Materials and methods

This single-blind randomized clinical trial was approved by the Medical Ethics Committee at Hamadan University of Medical Sciences in Hamadan, Iran (IR.UMSHA.REC.1397.725), and was registered in the Iranian Registry of Clinical Trials (IRCT20190106042253N1).

A total of 120 orthodontic patients between 15 and 25 years who started their fixed orthodontic treatment at the Orthodontics Department of Hamadan University of Medical Sciences from February 2019 to October 2019 participated in this study.

The inclusion criteria were (1) patients who received their orthodontic appliances within 12 weeks after starting the study, (2) patients without any mental and physical disability or craniofacial disorders, (3) patients who had smartphones with Android version ≥ 4.0.3 or IOS version ≥ 9 operating systems, (4) patients without enamel or dentin dysplasia, (5) patients not taking medications affecting plaque accumulation such as antibiotics or antibacterial mouth rinses, and (6) patients without periodontal disease or dental caries at the study onset.

Patients were randomly divided into two groups of control (60 patients) and intervention (60 patients) by the balanced block randomization method. The sample size was calculated to be 60 in each group according to previous studies,^1,3^ assuming a power of 0.9, confidence interval of 95%, standard deviation (SD) of 0.5, and with the aim of finding a 0.3 difference in the groups’ means.

The patients assigned to the control group received oral hygiene instruction by the conventional method, comprising of explanation by a dentist, and utilizing instructive brochures and videos on oral hygiene compliance. Patients in the intervention group were educated to use a smartphone app, Brush DJ, in addition to the same conventional oral hygiene instruction as the control group by the same dentist. The app included timer and daily reminders, in order to assist patients to improve their oral hygiene status (the Brush DJ app was launched on the Apple App Store^11^ in November 2011 and on the Google Play Store^12^ in March 2012).^13^ The app has been signposted to members by the British Dental Association.^14^

Meanwhile, a questionnaire was given to all patients to evaluate the frequency and duration of brushing per day in both groups, and the frequency of app usage and reminder noticing in the intervention group.

Prior to the study onset, an informed consent was obtained from the participants. The outcomes were collected through clinical assessments and self‐administered questionnaires. The assessments were made at baseline, prior to the intervention (T0), and at 4 weeks (T1), 8 weeks (T2), and 12 weeks (T3) after the treatment onset (first treatment session).

All patients were given an Oral-B brochure for standardization of toothbrush, toothpaste, and dental floss, and they were asked to provide the same toothbrush and toothpaste in order to prevent instrumental bias. Also, the intervention group received instructions on how to use the app and its reminder settings. The Brush DJ app has been developed to encourage an oral hygiene routine. The app aims to motivate the users to brush for 2 min while listening to music. Also, it allows the users to set reminders to brush twice a day.^13^

In order to determine the patients’ plaque index (PI), a disclosing tablet (Svenska^®^, Svenska Dentorama AB, Solna Stockholm, Sweden) was used. For PI measurement, according to O’Leary plaque control record,^15^ the colored tooth surfaces after chewing the tablet were counted (except for occlusal surfaces) and divided by the total number of tooth surfaces.

The gingival index (GI), according to Loë and Sillnes^16^, was used to assess the severity of gingivitis. All four facial, lingual, mesial, and distal surfaces of the teeth were evaluated using a periodontal probe, and were graded from 0 to 3 (0: normal gingiva, absence of inflammation, bleeding, or swelling; 1: mild inflammation, oedema, slight discoloration, no bleeding; 2: inflammation, redness, moderate swelling, and bleeding on probing; 3: severe inflammation, redness, oedema, and spontaneous bleeding). The GI was measured for teeth #1.6, 2.1, 2.4, 3.3, 3.4, and 4.4,^1^ and before measuring the PI to prevent the probable bias by disclosing agent on GI score evaluation.

A general dentist, as the outcome evaluator, and the clinicians, who provided the orthodontic treatment, were blinded in order to prevent the observer bias. The process of app installation and instructions were made by an assistant who had no involvement in data collection or analysis.

Data were analyzed using SPSS version 21 (SPSS Inc., IL, USA). Statistical tests including the chi-square test, *t*-test, Pearson’s correlation test, and repeated measures ANOVA were utilized for data analysis. In case of violation of sphericity assumption of repeated measures ANOVA, the Greenhouse–Geisser test was applied and level of significance was set at 0.05.

## Results

**Fig. 1:**
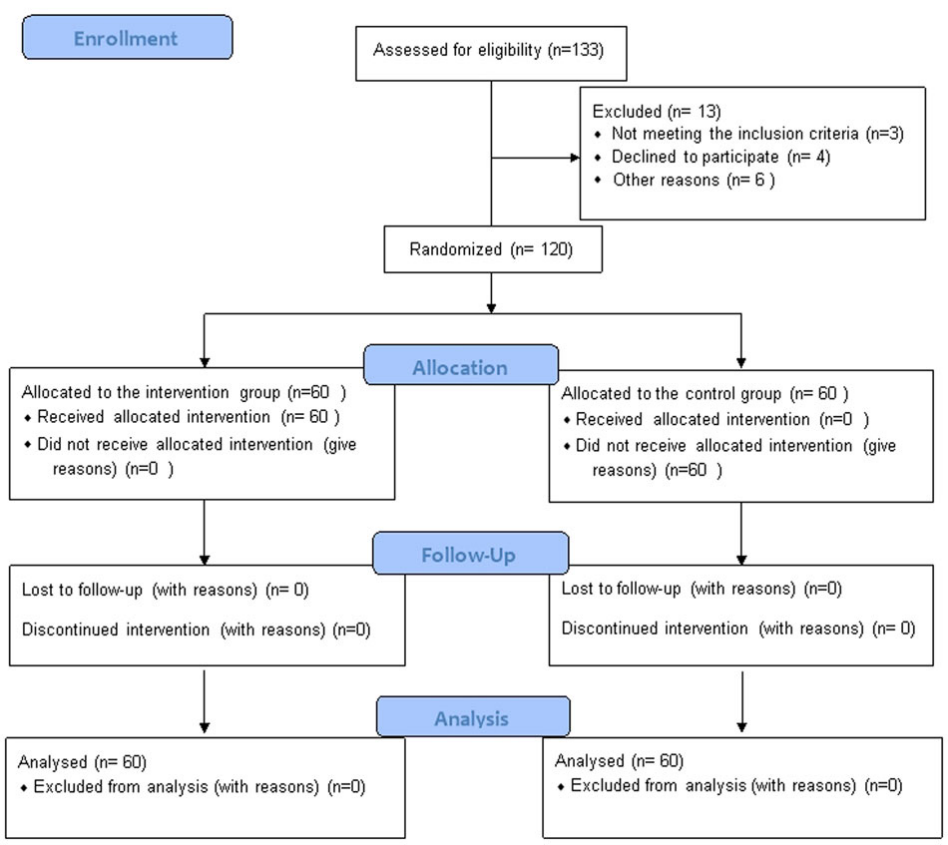
CONSORT 2010 patients’ flow diagram. Showing the number of cases involved in the two groups at different study periods.

As shown in Fig. 1, 120 out of 133 eligible patients with fixed orthodontic appliances signed an informed consent form, enrolled in the study, and were randomly divided into two groups: 60 patients in the intervention group (54 females and 6 males) and 60 patients in the control group (47 females and 13 males). Although a significant difference was noted in the number of females and males in each group (*p* < 0.001), a homogeneous distribution was shown in the two groups (*p* = 0.080). The mean age of patients at the onset of treatment was 18.7 ± 3.87 and 19.27 ± 3.65 years in the intervention and control groups, respectively. The difference in this respect between the two groups was not statistically significant (*p* = 0.406).

The PI and GI were assessed at baseline (T0), and at 4 weeks (T1), 8 weeks (T2), and 12 weeks (T3) after the first treatment session (Table 1).

**Table 1:** Mean PI in the two groups at different time points.

	Baseline (T0)	First follow-up (T1)	Second follow-up (T2)	Third follow-up (T3)	*p* value^a^	
	Mean ± SD	Mean ± SD	Mean ± SD	Mean ± SD	Intergroup comparison	Intragroup comparison
Intervention group	75.21 ± 13.36	73.39 ± 12.50	69.18 ± 11.84	67.84 ± 12.33	**p(GROUP): <0.001 **p(TIME × GROUP): <0.001	<0.001**
Control group	76.59 ± 12.76	76.89 ± 11.11	78.90 ± 8.89	80.82 ± 10.05		0.028

**p-values are statistically significant.

^a^RM-ANOVA.

Since the results of *t*-test for PI at baseline did not show a significant difference between the intervention and control groups, repeated measures ANOVA was used for intergroup comparisons.

**Fig. 2:**
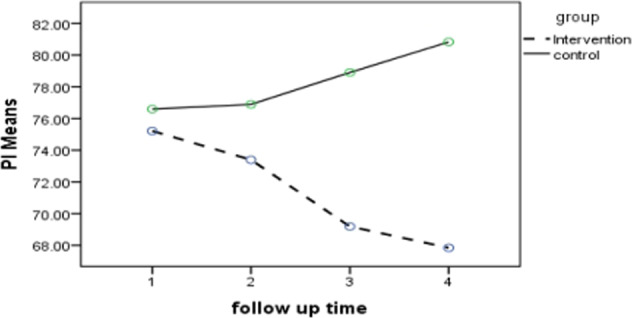
Plaque index differences between the two groups. Showing this index measures in the two groups at the baseline and three months of follow-ups.

The results of RM-ANOVA showed significant differences in PI changes between the two groups (*p* < 0.001). The interaction effect of group-time was also significant on PI (*p* < 0.001). As shown in Fig. 2, there was a reduction in plaque accumulation in the intervention group, while the plaque accumulation was significantly greater in the control group.

Regarding the significant difference in intragroup comparisons in both the intervention and control groups for PI changes during the follow-up period, the Bonferroni post hoc test was utilized to compare the changes in each group. As shown in Table 2, the PI decreased during the follow-up in the intervention group, and this reduction was significant at T2 (*p* = 0.021) and T3 (*p* = 0.003) in comparison with T0, and at T3 in comparison with T1 (*p* = 0.030). In contrast, this index increased over time in the control group, but this increment was not statistically significant at any time point.

**Table 2:** Intragroup pairwise comparisons of PI.

	Mean difference	*p* value^a^
Intervention group		
Baseline (T0)		
First follow-up (T1)	1.81	1
Second follow-up (T2)	6.02	0.021**
Third follow-up (T3)	7.37	0.003**
First follow-up (T1)		
Second follow-up (T2)	4.21	0.111
Third follow-up (T3)	5.55	0.030**
Second follow-up (T2)		
Third follow-up (T3)	1.34	1
Control group		
Baseline (T0)		
First follow-up (T1)	−0.3	1
Second follow-up (T2)	−2.31	0.813
Third follow-up (T3)	−4.24	0.119
First follow-up (T1)		
Second follow-up (T2)	−2.01	0.672
Third follow-up (T3)	−3.94	0.096
Second follow-up (T2)		
Third follow-up (T3)	−1.93	0.561

**p-values are statistically significant.

^a^Bonferroni post hoc test.

**Fig. 3:**
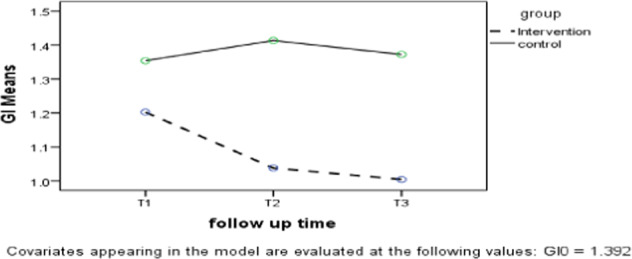
Gingival index differences between the two groups. Showing this index measures in the two groups at three months of follow-ups.

Since the results of *t*-test for GI at baseline showed a significant difference between the intervention and control groups, repeated measures ANCOVA was used for intergroup comparisons. In the intervention group, GI declined during the follow-up period, while in the control group there was an increase in the second follow-up (T2) in contrast with T1, and a reduction in the third follow-up (T3) in comparison with the second follow-up (T2; Fig. 3).

As shown in Table 3, significant differences were noted between the two groups in GI (*p* < 0.001). Also, the interaction effect of group-time on GI was significant (*p* < 0.001).

**Table 3:** Mean GI in the two groups over time.

	Baseline (T0)	First follow-up (T1)	Second follow-up (T2)	Third follow-up (T3)	*p* value^a^	
	Mean ± SD	Unadjusted mean ± SD (adjusted mean ± SD)	Unadjusted mean ± SD (adjusted mean ± SD)	Unadjusted mean ± SD (adjusted mean ± SD)		
Intervention group	1.29 ± 0.49	1.13 ± 0.52 (1.20 ± 0.04)	0.98 ± 0.44 (1.04 ± 0.04)	0.95 ± 0.43 (1.00 ± 0.05)	**p(GROUP): <0.001 **p(TIME × GROUP): <0.001	<0.001**
Control group	1.49 ± 0.59	1.43 ± 0.57 (1.35 ± 0.04)	1.47 ± 0.54 (1.41 ± 0.04)	1.43 ± 0.56 (1.37 ± 0.05)		0.378

**p-values are statistically significant.

^a^RM-ANCOVA.

The intragroup comparisons of GI revealed a significant difference only in the intervention group; thus, the Bonferroni post hoc test was used in order to compare the mean differences during the follow-up period. As shown in Table 4, there was a reduction in GI at each time point compared with its previous time point, and significant differences were noted at all-time points, except for the difference between T3 and T2 (*p* = 1).

**Table 4:** Intragroup comparisons of GI.

Intervention groups	Mean difference	*p* value^a^
Baseline (T0)		
First follow-up (T1)	0.16	0.029**
Second follow-up (T2)	0.31	<0.001**
Third follow-up (T3)	0.34	<0.001**
First follow-up (T1)		
Second follow-up (T2)	0.15	0.034**
Third follow-up (T3)	0.18	0.009**
Second follow-up (T2)		
Third follow-up (T3)	0.03	1

**p-values are statistically significant.

^a^Bonferroni post hoc test.

For easier comparisons, the mean values of frequency and duration of tooth brushing per month were calculated (Table 5). Comparison of the two groups revealed insignificant differences in brushing duration (*p* = 0.362) and frequency (*p* = 0.359). As shown in Table 5, at 2 months (T2), both brushing frequency and duration improved in the control group and decreased in the intervention group, while there was a reduction in brushing duration at T3 compared with T2 and T1 in the control group.

**Table 5:** Mean frequency and duration (in minutes) of tooth brushing in the two groups during the follow-up period.

	First follow-up (T1)	Second follow-up (T2)	Third follow-up (T3)	*p* value^a^	
	Mean ± SD	Mean ± SD	Mean ± SD	Intergroup comparison	Intragroup comparison
Brushing frequency					
Intervention group	1.88 ± 0.55	1.86 ± 0.45	1.87 ± 0.50	*p*(TIME): 0.283 *p*(GROUP): 0.359 *p*(TIME × GROUP): 0.150	0.089
Control group	1.98 ± 0.79	2.03 ± 0.74	1.90 ± 0.77		0.847
Brushing duration					
Intervention group	4.62 ± 2.93	4.33 ± 2.35	4.27 ± 2.39	*p*(TIME): 0.5 *p*(GROUP): 0.362 *p*(TIME × GROUP): 0.264	0.761
Control group	4.98 ± 4.74	5.09 ± 4.67	5.02 ± 4.97		0.122

^a^RM-ANOVA.

The app noticing frequency decreased at 2 months (1.29 ± 0.09) compared with 1 month (1.31 ± 0.08), which was related to brushing duration and frequency reduction. At 3 months, the frequency of attention to app reminder increased (1.31 ± 0.09).

As shown in Table 6, brushing frequency and duration were positively correlated with app usage during the follow-up period, although the correlation between brushing duration and app usage was not statistically significant at T1 (*p* = 0.214).

**Table 6:** Application usage frequency during the follow-up period.

Follow ups	Application usage frequency	Pearson’s correlation	
		*p* value^a^	
	Mean ± SD	Brushing duration	Brushing frequency
First follow-up (T1)	1.31 ± 0.08	*r* = 0.16	*r* = 0.55
		*p* = 0.214	*p* < 0.001**
Second follow-up (T2)	1.29 ± 0.09	*r* = 0.26	*r* = 0.51
		*p* = 0.043**	*p* < 0.001**
Third follow-up (T3)	1.31 ± 0.09	*r* = 0.29	*r* = 0.50
		*p* = 0.024**	*p* < 0.001**

**p-values are statistically significant.

^a^Pearson’s correlation test.

The age of participants in this study was 15–25 years. They were divided into two groups of 15–18 (34 patients in the intervention group and 29 patients in the control group) and over 18 years (26 patients in the intervention group and 31 patients in the control group). The age distribution of patients was normal in the both intervention and control groups.

In the intervention group, the frequency of application noticing in 15–18 year-olds was higher than that in over 18 years old (Table 7).

**Table 7:** Correlation of different age groups with app usage frequency, brushing duration, and brushing frequency

		First follow-up (T1)		Second follow-up (T2)		Third follow-up (T3)	
	Age group	15–18	>18	15–18	>18	15–18	>18
App usage frequency	Intervention group	1.50 ± 0.54	1.05 ± 0.66	1.45 ± 0.67	1.08 ± 0.66	1.44 ± 0.69	1.13 ± 0.7
	*P*-value*	0.005**		0.039**		0.092	
Brushing frequency	Intervention group	1.96 ± 0.52	1.76 ± 0.58	1.86 ± 0.44	1.85 ± 0.47	1.87 ± 0.45	1.87 ± 0.57
	*P*-value*	0.170		0.965		0.985	
	Control group	2.06 ± 0.92	1.9 ± 0.66	2.17 ± 0.96	1.9 ± 0.42	2.09 ± 0.96	1.73 ± 0.48
	*P*-value*	0.439		0.164		0.176	
Brushing duration	Intervention group	4.06 ± 1.33	5.34 ± 4.11	3.81 ± 1.04	5 ± 3.28	3.89 ± 1.02	4.76 ± 3.41
	*P*-value*	0.092		0.051		0.165	
	Control group	6.03 ± 6.04	3.99 ± 2.85	6.17 ± 5.97	4.08 ± 2.73	6.27 ± 6.32	3.85 ± 2.89
	*P*-value*	0.098		0.084		0.059	

*Independent *t*-test.

**p-values are statistically significant.

## Discussion

In this randomized clinical trial, the effectiveness of a smartphone app for improvement of orthodontic patients’ oral hygiene status was investigated. We selected the Brush DJ app (which is a reminder app and it is the only approved dental app for inclusion in the NHS apps library: https://www.nhs.uk/apps-library/brush-dj/) to motivate oral hygiene routine, and help patients manage their tooth brushing frequency and duration. This app aims to prompt users to brush for 2 min while listening to music, chosen either from a playlist, or randomly, from the music stored in the user’s device or music streaming service. Moreover, this app allows the users to set reminders to brush twice a day. The app also links to videos published on YouTube^17^ showing how to effectively use a toothbrush, dental floss, or interdental brushes.^13^

In order to assess the efficacy of the Brush DJ app for improvement of the patients’ oral hygiene compliance, the app usage frequency, brushing duration, and brushing frequency were assessed by a self-reported questionnaire during 90 days. Additionally, the GI and PI were measured at baseline (T0), and at 4 weeks (T1), 8 weeks (T2), and 12 weeks (T3) later to evaluate the participants’ oral hygiene status. The data collection procedure was similar in both groups, except that the control group’s questionnaire did not include the “app usage frequency” part.

Regarding the sex distribution in this study, the number of participating females was greater than males, as more females usually seek orthodontic treatment in all age groups except 40 years old and over.^18^

In the present study, significant differences were noted in PI and GI changes between the two groups (*p* < 0.001). A reduction in PI and GI in the intervention group was noted in comparison with the control group.

Although some previous studies used smartphone apps as instructional tools,^10,19^ others have utilized these apps as reminders.^5,20^ Many studies have reported results similar to ours: Zotti et al.^1^ assessed the efficacy of a WhatsApp-based program that was a combination of oral hygiene maintenance during orthodontic treatment and using a chat room known as “Brush Game.” Their results indicated the effectiveness of the intervention for improving both the oral hygiene and oral health of adolescents with fixed appliances. Patients, who had participated in the chat room, acquired significantly lower PI and GI values.

Eppright et al.^3^ sent a reminder text message each week to parents of patients in the text message group. The modified GI and PI scores were significantly lower in the text message group than in the control group at the fourth appointment after baseline. In another study, a mobile app was designed by Alkadhi et al.^21^ This app consisted of oral hygiene instructional videos and text messages, which intended to encourage patients to practice oral hygiene routines. This study showed that the app decreased the PI and GI more effectively than verbal oral hygiene instructions. Scheerman et al.^5^ developed the “WhiteTeeth” app to help adolescents with fixed orthodontic appliances performing their oral self-care behavior.^22^ The app was given to the intervention group in addition to usual care, while the control group only received the usual care.^5^ At the 6-week follow-up, the intervention led to a significant decrease in gingival bleeding. At the 12-week follow-up, dental plaque accumulation and the number of sites covered with plaque decreased significantly more in the intervention group than in the control group. In another study by Cozzani et al.^23^, patients were divided into three groups: the first group did not receive any post-procedure communication (control group), the second group received a structured encouragement text message, and the third group received a structured phone call. Both the text message and phone call groups showed lower PI than the control group. In addition, Li et al.^20^ divided the participants into two groups of WeChat group (received regular reminders and educational messages) and control group. In their study, orthodontic PI and modified gingivitis index did not show statistically significant differences between the groups, which was in contrast to our results. The reason for the difference in the results can be attributed to the app format and usage in their study compared with our study.

Comparison of the mean brushing duration and frequency between the two groups as reported by individuals revealed insignificant differences. Similarly, Scheerman et al.^5^ found no significant effect of intervention on the oral health behavior score, tooth brushing (frequency and duration), or interproximal brush usage. We assumed that these results are due to the inaccurate reports of patients regarding the frequency and duration of brushing. The reduction in plaque accumulation in the app user group may reflect changes in the brushing technique in the intervention group.

In the present study, the frequency of application noticing in 15–18 years old was greater than that in over 18 years old. We believe that the probable reason is the fact that 15–18-year-old adolescents are more enthused about the apps, and spend more time on utilizing smartphones. To our best knowledge, the relationship between age and oral hygiene compliance has not been investigated in previous studies.

One of the most important limitations of the present study was that the app noticing frequency, brushing duration, and brushing frequency all depended on the individuals’ self-reports. It would be ideal to design apps with greater control over the duration and frequency of tooth brushing via a Bluetooth connection with the toothbrush or sound detection sensors that detect and record the brushing position, and options for sharing oral care activity with a dental care provider^5^ and use them to promote the oral health of orthodontic patients in future studies. However, it should be mentioned that these technologies ought to be added to electronic brushes, which are more expensive and hazardous for environment, and this can be a preventive factor itself.

## Conclusion

Our study indicated that PI and GI decreased in the intervention group who used the Brush DJ app in comparison with the control group. In addition, the app usage frequency was positively correlated with the brushing duration and frequency.

We believe that smartphone apps, as motivators and reminders, can improve orthodontic patients’ oral hygiene compliance, especially in adolescents.
